# Beyond the Antigen Receptor: Editing the Genome of T-Cells for Cancer Adoptive Cellular Therapies

**DOI:** 10.3389/fimmu.2013.00221

**Published:** 2013-08-05

**Authors:** Angharad Lloyd, Owen N. Vickery, Bruno Laugel

**Affiliations:** ^1^Institute of Infection and Immunity, Cardiff University School of Medicine, Cardiff, Wales, UK

**Keywords:** T-cells, genome editing, cancer, cell therapies, immune checkpoints

## Abstract

Recent early stage clinical trials evaluating the adoptive transfer of patient CD8^+^ T-cells re-directed with antigen receptors recognizing tumors have shown very encouraging results. These reports provide strong support for further development of the therapeutic concept as a curative cancer treatment. In this respect combining the adoptive transfer of tumor-specific T-cells with therapies that increase their anti-tumor capacity is viewed as a promising strategy to improve treatment outcome. The *ex vivo* genetic engineering step that underlies T-cell re-direction offers a unique angle to combine antigen receptor delivery with the targeting of cell-intrinsic pathways that restrict T-cell effector functions. Recent progress in genome editing technologies such as protein- and RNA-guided endonucleases raise the possibility of disrupting gene expression in T-cells in order to enhance effector functions or to bypass tumor immune suppression. This approach would avoid the systemic administration of compounds that disrupt immune homeostasis, potentially avoiding autoimmune adverse effects, and could improve the efficacy of T-cell based adoptive therapies.

## Introduction

Although there is still controversy over the role of the immune system in protecting the organism against the development of neoplasms in a natural setting (1) it is well accepted that artificial immunity can efficiently contain and even eradicate established tumors (2). Harnessing the anti-tumor potential of T-cells, and particularly CD8^+^ T-cells, is a promising approach for curative cancer treatment. Because of their relative ease of administration and documented low toxicities therapeutic vaccines that trigger T-cell responses are a very attractive approach. However, even though they efficiently induce antigen-specific immunity, the clinical benefit of cancer vaccines has so far been limited (3). In contrast adoptive cell therapies (ACT), where T-cells are modified *ex vivo* and re-infused in a patient’s circulation, are more difficult to implement and require important infrastructural investment. Yet a number of studies have now reported long-term remissions or tumor clearance (4–6), warranting further development of the therapeutic concept.

While conferring the immune system with the ability to recognize tumors through vaccination or ACT is a pre-requisite for the induction of efficient anti-tumor responses it is likely insufficient to achieve long-term clinical benefit in a majority of patients. An increasing body of evidence points to the necessity of combining different therapeutic approaches in order to improve treatment outcome (7, 8). For instance several small-molecule compounds that target oncogenic pathways also enhance tumor destruction by immune mechanisms, e.g., by sensitizing cancer cells to cytolysis (9, 10). The coordinated delivery of these compounds with immunotherapies is expected to improve clinical responses in an additive or even synergistic manner. Similarly the combination of immune-based therapies also holds great potential. Monoclonal antibodies (mAbs) blocking immune checkpoint receptors have recently emerged as promising therapeutics and many believe that the recent marketing authorization of Ipilimumab, targeting CTLA-4, heralds great strides in this area. Immune checkpoint receptor blocking agents are currently marketed or developed as single therapies but are expected to achieve maximal efficacy in combination with immune stimulatory approaches such as vaccination or ACTs (11, 12). Although generic treatment combinations will undoubtedly provide some degree of clinical benefit it is the prospect of developing personalized therapies tailored to individual needs that holds the greatest potential to improve clinical outcome in cancer therapy. The heterogenous nature of similar tumor histologies as well as individual genetic variability are believed to account for the varied response levels to generic treatments and the wider availability of prognostic tools should help define adequate treatment options that improve patient response. With respect to cancer vaccines or ACTs information about the nature of the immune checkpoint pathway(s) relevant to a tumor would be particularly useful in order to counteract immune suppression.

T-cell-based ACTs rely on the infusion in a patient’s circulation of *ex vivo* expanded tumor-infiltrating lymphocytes (TILs) or peripheral blood T-cells transduced with viral vectors expressing a tumor-specific antigen receptor. This engineering step offers the opportunity to transfer additional genetic material conferring T-cells with enhanced anti-tumor activity. Targeted genome editing relying on viral gene transfer could readily be combined with the delivery of antigen receptors at little additional cost in one unique therapeutic entity. This approach would avoid the drawbacks associated with combining treatment modalities of different nature requiring distinct administration regimens, e.g., cellular therapy and mAb injection. In addition cell-intrinsic disruption of immune checkpoints in tumor-specific T-cells is likely to display a better safety profile than the systemic administration of blocking agents. Recently developed gene targeting technologies such as zinc-finger proteins (ZFPs), transcription activator-like proteins (TALs), and RNA-guided endonucleases (RGENs) could thus be harnessed in order to silence the expression of T-cell-intrinsic genes that restrain their anti-tumor potential.

## Main Text

### Technical aspects and challenges to the modulation of gene expression in T-cells

RNA interference (RNAi) often is the technique of choice to silence gene expression in somatic cells and lentivirus-mediated RNAi is a good option for sustained and efficient silencing. Most lentiviral RNAi systems express short-hairpin RNAs (shRNAs) from RNApolIII promoters, which drive high levels of transcription using precise initiation and termination sites. A recurrent problem of lentivirus-mediated RNAi, which is particularly salient in T-cells (13), is that the constant generation of shRNAs interferes with endogenous miRNA biogenesis and can result in the deregulation of gene expression (14, 15). This issue has prompted investigators to seek alternative methods to silence gene expression (16).

**Figure 1:**
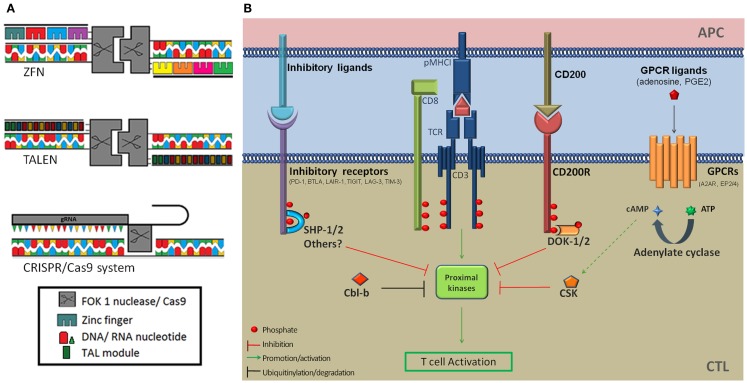
**(A)** Schematic diagram of the ZFN, TALEN, and CRISPR/Cas9 genome editing tools. **(B)** Inhibition of T-cell activation by immune checkpoint receptors and downstream signaling proteins. Several co-inhibitory receptors (PD-1, BTLA, and LAIR-1) inhibit T-cell signaling by recruiting the SHP-1 and/or SHP-2 tyrosine phosphatases at proximity of the TCR signaling complex via ITIMs and ITSMs. This results in the dephosphorylation of proximal kinases downstream of TCR triggering. In addition PD-1 ligation was shown to induce increased expression of the Cbl-b E3 ubiquitin ligase, which targets signaling molecules for degradation. Activation of the CD200R leads to the recruitment of DOK2 and RasGAP to its intra-cellular domain, resulting in the inhibition of downstream MAP kinases. The adenosine receptor 2A and PGE2 receptors EP2 and EP4 modulate T-cell activation through mobilization of the cAMP-PKAI-CSK pathway. CSK phosphorylates the inhibitory C-terminal tyrosine residue of Src kinases and negatively regulates TCR signaling. A2AR, adenosine A2a receptor; APC, antigen-presenting cell; BTLA-4, B- and T-lymphocyte attenuator; Cbl-b, casitas B-cell lymphoma; CTL, cytotoxic T-lymphocyte; DOK-1/2, docking protein 1/2; EP2/4, prostaglandin E receptor 2/4; Erk, extra-cellular signal regulated kinases; ITIM, immunoreceptor tyrosine-based inhibition motif; ITSM, immunoreceptor tyrosine-based switch motif; GPCR, G-protein coupled receptor; LAG-3, lymphocyte-activation gene 3; LAIR-1, leukocyte-associated immunoglobulin-like receptor 1; Lck, lymphocyte-specific protein tyrosine kinase; MHC1, major histocompatibility complex class 1; PD-1, programed death receptor 1; PD1-L1, programed death receptor 1-ligand 1; SHP-1, Src homology 2 domain containing protein tyrosine phosphatase; TCR, T-cell receptor; TIGIT, T-cell immunoreceptor with Ig and ITIM domains; TIM-3, T-cell immunoglobulin domain and mucin domain 3.

Recently developed genome editing technologies based on DNA-targeting proteins have the potential to revolutionize ACTs by offering convenient tools to alter gene expression. TAL effector-nucleases (TALENs) and ZFP-nucleases (ZFNs) effect complete gene knockout (Figure 1A) and are promising alternatives to RNAi for therapeutic applications (17–19). TALs are bacterial DNA-binding proteins consisting of near identical 34 amino-acid modules that bind one nucleotide with high affinity. The variable 12th and 13th amino-acids of TALs, called repeat-variable di-nucleotide confers base specificity (NN → G/A, NI → A, NG → T, NK → G, HD → C, and NS → A/T/C/G) and TAL arrays that target a nucleotide sequence can be generated by assembling individual modules (17, 20). ZFPs are eukaryotic DNA-binding proteins. Cys2-His2 fingers, which are used for genome editing, are the most common ZFP motif (21) and are each specific for a nucleotide triplet. Artificial ZFP domains that target specific DNA sequences, usually 9–18 nt long, can be constructed by assembling individual fingers (18). ZFPs and TALs have similar modular configurations but TALs can in theory target any stretch of nucleotides beginning with a thymidine whereas some structural incompatibilities between individual ZFP modules due to overlapping DNA-binding domains make the assembly of oligomeric ZFPs error-prone and narrow down the diversity of possible target DNA sequences. A successful and popular application of these technologies is the fusion of customized ZFPs or TALs to the catalytic domain of the restriction nuclease Fok1 (ZFNs and TALENs). Fok1 nucleases catalyze DNA double strand breaks (DSBs) when they dimerize (22). TALENs and ZFNs are therefore designed in pairs that target adjacent sequences on opposite DNA strands, thereby promoting Fok1 dimerization, separated by a spacer region where DNA cleavage occurs. Non-homologous end joining (NHEJ) repair of DSBs results in insertions or deletions at the DNA cleavage site (17, 23) and bi-allelic frameshift mutations that result in complete knockout occur at low frequencies (18, 24). The overall efficiency of the approach is sufficient to generate knockout cells following appropriate selection procedures. Of note TALEN design is more flexible as they can accommodate spacers of different lengths (25) whereas ZFNs strictly require 5–7 nt (26). Taking this into account, as well as structural constraints mentioned above, it is estimated that the frequency of target sequences is 1 in 500 bp for ZFNs and 1 in 35 bp for TALENs (20).

RNA-guided endonucleases provide a distinct and attractive alternative to genome editing compared with protein-guided nucleases. The functions of clustered regularly interspaced short palindromic repeats (CRISPR) and CRISPR-associated (Cas) proteins as a system providing adaptive immunity to bacteria against bacteriophages (27, 28) was recently harnessed for genome engineering (29, 30). The Cas9 nuclease binds to a short complementary RNA (crRNA) providing DNA-targeting specificity and to a trans-activating crRNA (tracRNA), required for crRNA processing, expressed individually or combined as a chimeric guide RNA (gRNA) (Figure 1A). CRISPR-Cas9 systems displayed a cleavage efficiency comparable (31) or superior (32) to TALENs in human cells. The clear advantage of RGEN is that it can be easily adapted to target different genomic sequences by customizing the synthetic crRNA/gRNA delivered in combination with Cas9 (33). In comparison ZFNs and TALENs require extensive engineering and validation steps.

The delivery of genome editing agents to T-cells is a crucial aspect of their successful application to ACTs. Because nuclease-based genome editing relies on generating transmissible mutations, protein- or RNA-guided nucleases only need to be transiently expressed. In fact transient expression probably minimizes off-target DNA cleavage (34). Provasi et al. have used integration-deficient lentiviruses as well as adenoviruses in order to modify the genome of T-cells with ZFNs (35). Of note it was recently shown that, due to their very repetitive nature, TAL arrays were incompatible with efficient reverse transcription required for the delivery of genetic material using lentiviruses (36), thereby limiting the range of delivery methods for TALENs.

### Application of therapeutic genome editing to T-cells

Crucially therapies based on T-cell genome editing have already entered clinical development. A phase II clinical trial based on preventing the expression of the CCR5 gene, acting as a co-receptor for HIV in CD4^+^ T-cells, using ZFNs (37) was recently initiated for the treatment of HIV/AIDS (NCT01252641). The safety results will be of huge importance for ZFN-based therapies and for genome editing in T-cells in general. Moreover a recent study provided proof of concept for the combination of TCR gene delivery with genome editing by using ZFNs specific for the endogenous constant TCR gene segments in order to prevent mispairing with ectopic TCR chains (35). The success of this approach provides a good rationale for wider applications of ZFN genome editing to T-cells.

Enhancing the anti-tumor potential of CD8^+^ T-cells through genome editing can be done in many ways. Here we will focus on disrupting the expression of genes that inhibit T-cell functions as a result of the suppressive activity of the tumor micro-environment. T-cell inhibitory pathways targeted by genome editing in the context cancer ACTs should meet several criteria. First, their mechanism of action should be strictly cell-intrinsic. Second, they should be relevant to effector T-cells as opposed to naive T-cells. For instance CTLA-4 does not meet these two criteria since it works at least partly by reducing the availability of co-stimulatory molecules on the surface of antigen-presenting cells during the priming of naive T-cells (38). Finally, since only anti-tumor T-cells are modified, one of the advantages of this approach is that it allows targeting ubiquitous suppressive pathways whose systemic blockade or inhibition might result in serious adverse effects. Because it is clinically validated the most obvious target is probably PDCD1: the gene encoding the co-inhibitory receptor PD-1. PD-1 is expressed on activated T-cells and its engagement by its two known ligands PD-L1 and PD-L2 inhibits proximal signaling events triggered by TCR stimulation through recruitment of the phosphatase SHP-2 (39) and increased expression of the E3 ubiquitin ligase Cbl-b (40), which impair key components of the TCR signaling cascade through dephosphorylation and proteasomal degradation (Figure 1B). High cellular expression levels of PD-1 are characteristic of exhausted CD8^+^ T-cells in chronic viral infections as well as TILs and correlate with impaired effector functions (41). Histological analyses have revealed that numerous tumor types express one or both PD-1 ligands (42, 43), prompting the targeting of this pathway in order to augment anti-tumor immunity. PD-1 blockade has shown promising objective response rates in a range of cancer indications and it is anticipated that PD-1 blocking agents will be approved for marketing authorization as mono-therapies. In addition these therapeutics are evaluated in combination with cancer vaccines, small-molecule signaling inhibitors, tumor-targeting mAbs, and cytokine therapy (http://clinicaltrials.gov/ct2/results?term=pd1&Search=Search). The combination of PD-1 blockade with these treatments, as well as with cancer ACTs, is expected to further enhance anti-tumor activity (11). Several other co-inhibitory receptors expressed by T-cells qualify as targets for gene editing coupled with antigen receptor delivery (Table 1; Figure 1B). *In vivo* and *in vitro* pre-clinical data strongly support the development of reagents targeting TIM-3 and LAG-3. Dual targeting of PD-1 and TIM-3 or LAG-3 with mAbs synergistically enhanced anti-tumor responses (44) and pre-clinical evaluations of a soluble Fc-LAG3 complex, which has now entered clinical development, were promising (45). Other targets are currently under similar evaluation procedures and might expand the list of druggable co-inhibitory receptors for cancer immunotherapy (Table 1; Figure 1B).

**Table 1:** **Potential immune checkpoint receptor targets for genome editing in the context of cancer adoptive cellular therapies**.

	Name (gene)	Function	Ligand	Intra-cellular signaling/second messengers	Recognition motif	References
Co-inhibitory receptors	PD-1 (CD279)	Inhibition of T-cell activation and promotion of tolerance	PD-L1 (B7-H1)	SHP-1	ITIM	Keir et al. (56), Parry et al. (57)
			PD-L2 (B7-DC)	SHP-2	ITSM	
	LAG-3 (CD223)	Down regulation of T-cell cytokine secretion and proliferation	MHCII	–	–	Pardoll (12), Turnis et al. (58)
	BTLA (CD272)	Suppression of T-cell response	HVEM	SHP-2	ITIM	Murphy et al. (59), Watanabe et al. (60)
	OX2R (CD200R)	Inhibits T-cell function	CD200	DOK2	NPxY	Kretz-Rommel et al. (61), Moreaux et al. (62), Pallasch et al. (63)
	TIM-3	Down regulation of T-cell cytokine secretion and proliferation	Galectin 9	–	–	Pardoll (12), Zhu et al. (64)
			Phosphatidylserine	
	TIGIT	Inhibition of T-cell activation	VR, PVRL2, and PVRL3	–	ITIM	Joller et al. (65)
	LAIR-1	Inhibits cytotoxic activity	Collagen	SHP-1	ITIM	Lebbink et al. (66), Meyaard (67)
				SHP-2	
Receptors for soluble regulatory mediators	PGE2 receptors EP2/4	Inhibition of T-cell activity	PGE2	Adenylyl cyclase	–	Mahic et al. (68), Oberprieler et al. (53)
				cAMP	
	Adenosine receptor 2A (A2AR)	Blocks T-cell activity	Adenosine	Adenylyl cyclase cAMP	–	Pardoll (12), Ohta et al. (50), Raskovalova et al. (69)

*Non-extensive list of immune checkpoint receptors known to impair anti-tumor T-cell immunity in a cell-intrinsic manner. The relevant intra-cellular signaling and second messenger pathways, when known, are indicated*.

The presence of cognate ligands within the tumor micro-environment is a crucial aspect for targeting co-inhibitory receptors and other immune checkpoint receptors. In the case of PD-1 retrospective analysis of patient biopsies in the phase Ib clinical trial assessing the blocking mAb BMS-936558 showed that the objective response rate in patients whose tumors expressed PD-L1 reached 36% compared with 18% in the entire cohort and 0% among patients with PD-L1-negative tumors (46). These striking results highlight the importance of prognosis and patient stratification for the design of appropriate cancer immunotherapies based on PD-1 inhibition. Such a strong correlation is still to be established for other immune checkpoint receptors but it is tempting to speculate that similar principles are applicable. However, even though their relevance in tumor immunity is established, it is not entirely clear what the actual ligands for several co-inhibitory receptors are in the context of anti-tumor immunity. More fundamental and clinical investigations are required in order to unambiguously identify relevant inhibitory ligands and assess their presence in the tumor environment.

Several soluble regulatory mediators also act as immune checkpoints in anti-tumor immunity. For instance high levels of extra-cellular adenosine are found in the vicinity of many solid tumors because of the hypoxic environment, a well-known environmental factor promoting adenosine release. Suppressive adenosine is also generated through direct dephosphorylation of extra-cellular adenosine nucleotides by the cell-surface nucleotidases CD39 and CD73 expressed by regulatory T-cells (T_regs_) and some tumors, e.g., ovarian carcinomas (47, 48). The adenosine receptor 2A (A2AR), belonging to the G-protein coupled receptor family (GPCRs), is expressed on T-cells and has been identified as a target for immunotherapy for over a decade (12, 49–51). The A2AR inhibits T-cell activation through the cAMP-PKAI-CSK pathway (Figure 1B) and pre-clinical *in vivo* models have shown that A2AR knock-down or antagonism in adoptively transferred T-cells dramatically increased anti-tumor immunity (50). Inhibiting adenosine-mediated immune suppression is therefore believed to be an efficient strategy for cancer immunotherapies. Yet because adenosine receptors are ubiquitously expressed and involved in many physiological processes, especially in neurotransmission, classical antagonistic approaches are likely to result in a number of side effects. Such systemic adverse effects could be avoided in the context of adoptive T-cell therapies through T-cell-intrinsic gene editing or the *in vitro* selection of desensitized and irresponsive T-cells (52). Similarly, prostaglandin E2 (PGE2) directly suppresses T-cell activation through the cAMP second messenger pathway in effector/memory CD8^+^ T-cells (53). Tumor-associated T_regs_ as well as colorectal cancer cells express high levels of immunosuppressive PGE2 (54). Interfering with EP2/EP4 receptors expression in T-cells may therefore enhance their anti-tumor potential.

A broader, possibly riskier, alternative to targeting individual immune checkpoint receptors would be interfering with the expression of downstream molecules conveying intra-cellular inhibitory signals. For instance several co-inhibitory receptors use the tyrosine phosphatases SHP-1 and/or SHP-2 to inhibit T-cell activation (Figure 1B). Inhibition of SHP-1/2 expression may therefore confer resistance to several checkpoint pathways used by tumors. Stromnes and colleagues reported that conditional knockout of SHP-1 in mature murine CD8^+^ T-cells improved effector cell functions and tumor clearance in an adoptive transfer setting similar to cancer ACTs without resulting in autoimmune toxicity, thereby providing a good rationale for such an approach (55).

## Concluding Remarks

T-cell based ACTs that rely on the re-infusion of patient T-cells expressing an artificial antigen receptor is an epitome of personalized medicine. These therapies require the identification of specific tumor antigens and/or patient HLA-type and would undoubtedly benefit from further prognostic analysis and subsequent treatment customization. Based on recent successes in cancer immunotherapy, immune checkpoint receptors that suppress T-cells represent a particularly attractive class of targets for such an approach. We believe that enhancing the anti-tumor potential of re-directed T-cells by targeting inhibitory pathways through genome editing can further improve the efficacy of cancer ACTs. Moreover cell-intrinsic inhibition of these pathways may display an advantageous safety profile compared with immune checkpoint blockade relying on the systemic administration of mAbs, recombinant proteins, or small molecules.

The field of genome editing is currently buzzing with new ideas and technologies that make the application of targeted gene knockout and gene correction a tantalizing prospect for the development of customized ACTs in regenerative medicine and immunotherapy. However, numerous potential pitfalls must be assessed in pre-clinical development. At this point ZFNs are the most advanced technology for genome editing and have already entered clinical development. However, their complicated and labor-intensive design, construction, and validation are major drawbacks for their widespread use in non-specialized laboratories, which often rely on commercial reagents. TALENs, on the other hand, are much more user-friendly and several toolboxes that allow investigators to generate custom reagents are available at low cost. With regard to T-cell based cancer ACTs, however, the incompatibility of TALENs with retroviral delivery, often used to express ectopic TCRs or CARs, might be problematic. Finally, CRISPR-Cas9 genome editing may prove to be the holy grail of genome editing but the current lack of hindsight on this technology does not allow concluding on its use yet. Thorough assessment of the respective advantages and drawbacks of each technology as well as pre-clinical feasibility and safety studies are warranted to validate genome editing applied to cancer ACTs.

## Conflict of Interest Statement

The authors declare that the research was conducted in the absence of any commercial or financial relationships that could be construed as a potential conflict of interest.
